# The Acid-Base/Deprotonation Equilibrium Can Be Studied with a MicroScale Thermophoresis (MST)

**DOI:** 10.3390/molecules27030685

**Published:** 2022-01-21

**Authors:** Paweł Mateusz Nowak, Michał Woźniakiewicz

**Affiliations:** Department of Analytical Chemistry, Faculty of Chemistry, Jagiellonian University, Gronostajowa 2, 30-387 Krakow, Poland; michal.wozniakiewicz@uj.edu.pl

**Keywords:** acid-base equilibrium, fluorescein isothiocyanate, fluorescence, MicroScale thermophoresis, thermal diffusion, thermodynamics

## Abstract

MicroScale thermophoresis (MST) is a rapidly developing bioanalytical technique used routinely for the examination of ligand-target affinity. It has never been used so far for the analysis of acid-base dissociation and the determination of p*K*_a_ constant. This work is the-proof-of-concept of this new idea. It demonstrates that the p*K*_a_ values obtained from the thermophoretic data are consistent with the reference methods. As a result, the analytical potential and utility of the MST technology can become even greater, especially if the new detection system of thermophoretic movement will be developed in the future. Even now, taking into account the necessity to use fluorescence, the proposed method may be useful in many respects.

## 1. Introduction

The MicroScale Thermophoresis (MST) technique has been developing dynamically for over a decade, gaining more and more recognition among biophysicists, biochemists and molecular biologists working at universities, as well as among representatives of research and development laboratories in the pharmaceutical industry [1]. Its major purpose is to test the affinity of non-covalent ligand-target interactions, such as drug-receptor, protein-protein, protein-ligand, DNA/RNA-ligand, and others. In addition, its applications are known to study the stoichiometry, stability and kinetics of ligand-target and enzymatic reactions [2,3,4,5,6,7,8].

The basic principle of the MST technique is to heat a small zone of the sample placed in a capillary kept in a dynamic equilibrium, with the use of an IR laser, to create a temperature gradient of about 5–10 K. The result is a thermophoresis process, i.e., thermal diffusion manifested by the migration of molecules present in the solution between a cold and warm zone (in most known cases it is the movement towards a cooler zone). The system thus tends to reach a new equilibrium characterized by different concentrations of molecules in the hot and cold zone. This ratio depends on the balance between thermophoresis (thermodiffusion) and ordinary diffusion, expressed by the Soret coefficient [9]. The model proposed by Duhr and Braun assume the dependence of Soret coefficient on the parameters characteristic for a given molecule, like hydrodynamic area, effective surface charge density and hydration entropy, which are directly related to molecular size, ionization and conformation. As these parameters change as a result of ligand-target interactions; the analysis of the thermophoretic response provides information on the degree of binding and affinity of the system. It is carried out based on the monitoring of fluorescence from one of the interacting molecules labeled with an appropriate fluorophore or showing native fluorescence. The observed signal is the change in intensity of fluorescence over time (MST response), including the stage before turning on the IR laser (cold), after turning the laser on (hot), and after turning it off again when the system returns to its initial state (cold). A single measurement usually takes about half a minute.

An important fact is that in addition to the thermophoresis phenomenon, the shape of the MST response is also influenced by the phenomena directly related to fluorescence, which are referred to as a temperature-related intensity change (TRIC) [1,2,3,4]. TRIC is most important at the beginning of the analysis, right after the IR laser is turned on, unlike thermophoresis, which manifests itself most at the later stage of collecting the signal. However, the response always depends on both effects which cannot be easily deconvoluted. The disadvantage is undoubtedly the detection method using fluorescence that often requires laborious fluorescent labeling of the target. There are, however, very strong advantages that make the MST technology more and more popular: high sensitivity, no need to immobilize the target, carrying out measurements in a solution with physiological composition, low sample consumption, and ease of use.

The idea behind the experiment described here was the completely new application of the MST technique for the analysis of acid-base dissociation, i.e., the deprotonation reaction that occurs under the influence of pH changes. Acid-base dissociation is accompanied by the change in charge and hydrodynamic radius of the molecule, although its molecular weight remains virtually unchanged. Taking into account the quadratic dependence of the Soret coefficient on the effective surface charge density and linear dependence on the hydrodynamic area [9], it was assumed that these could be sufficient premises to observe changes in the MST response of an analyte molecule having acid-base groups, caused by a change in the pH of the environment. Looking from the other side, it was assumed that an analyte molecule with the deprotonated group could play the role of a free target, the H^+^ ions a ligand whose concentration is expressed by pH value, and the protonated form of an analyte could play the role of a target-ligand complex. In further perspective, we wanted to find out whether the MST technique allows us to determine the acidity constant (p*K*_a_) and whether it will be close to the values obtained using alternative techniques. Acidity and the related ionization potential is also an extremely important parameter characterizing biomolecules, such as drugs, the pharmacokinetics and pharmacodynamics of which may directly depend on the p*K*_a_ value. Such a new application of the MST technique could significantly increase its analytical potential, making it even more competitive due to its multi-functionality.

The basic limitation that must be faced is the need to use fluorescence to monitor changes in concentration occurring as a result of thermophoresis. Two solutions are possible: (1) fluorescent labeling, which can be difficult to perform with small molecules and the fluorophore functional groups must not be able to show p*K*_a_ values in the tested pH range to avoid signal interference; or (2) the use of the natural fluorescent properties of the analyte, which would significantly limit the field of application of this method to natural fluorophores. For the purpose of this experiment, the latter solution was chosen because of its simplicity. The main intention was to test whether the MST response of a selected fluorescent dye with known acid-base properties changes with pH. It was also assumed that in the future new research technologies will be developed based on the phenomenon of thermophoresis, as it is only a matter of time before the bypassing of its greatest limitation, the need to use fluorescence. Hypothetically, if we rely on absorbance instead of fluorescence, the method would be much more universal (although much less sensitive). Therefore, this work is mainly focused on finding out how prospective this direction of research is. It is worthy of note that MST technology is still very young, and its dynamic development and growing appreciation in the environment should be a cause for optimism about the future and allow one to foresee new solutions.

**Figure 1:**
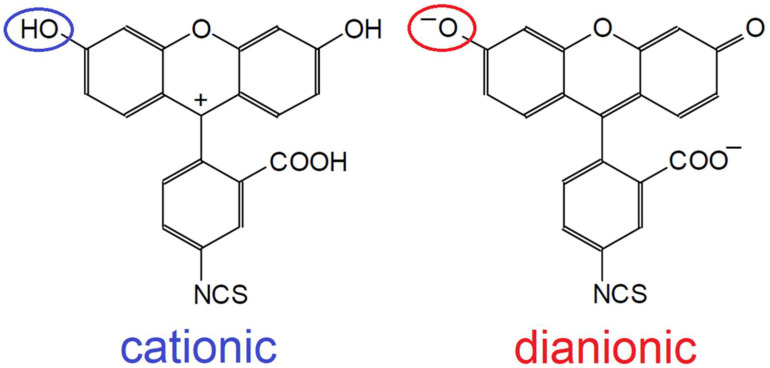
The cationic and dianionic forms of FITC and the phenolic group of interest.

Fluorescein isothiocyanate (FITC), a derivative of fluorescein widely used in a variety of bioanalytical methods, e.g., flow cytometry, was selected as the model fluorophore. FITC shows as many as three acid-base equilibria; it can exist in the forms ranging from cationic to dianionic, as shown in Figure 1. The p*K*_a_ values characterizing the individual equilibria are, according to the literature, 2.04, 4.36 and 6.62, respectively [10]. The last of them refers to the phenolic group, which, as a result of ionization, causes a rapid increase in fluorescence intensity depending on pH. The analysis of this equilibrium can therefore be easily performed with the fluorometric techniques, which can be a reference for the MST technique. The research was therefore carried out in the pH range covering the dissociation of this group, and the aim was to determine the corresponding p*K*_a_ value on the basis of the thermophoretic responses.

## 2. Results

### 2.1. Preliminary Investigation

**Figure 2:**
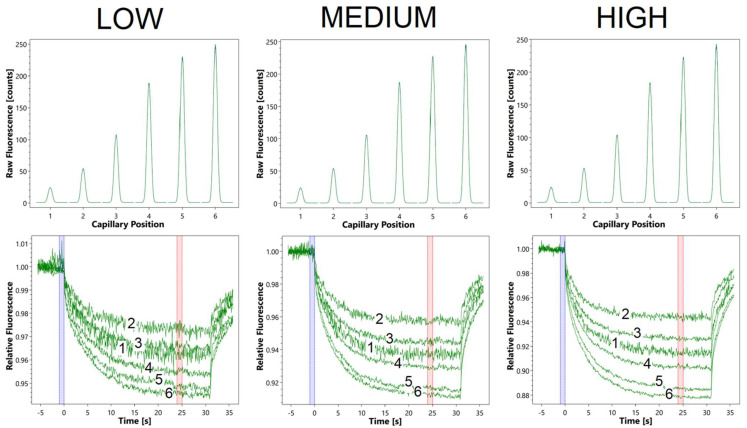
Data collected during the FITC analysis using the MST instrument; (**top**)—initial fluorescence intensity before turning on the IR laser for buffers with different pH: 1-pH 5.38, 2-pH 5.86, 3-pH 6.52, 4-pH 7.12, 5-pH 7.80, 6-pH 9.42; (**bottom**)—change of fluorescence over time from turning on the IR laser depending on pH, in the same buffers, with different IR laser powers: low, medium and high. The blue and red bars indicate the data used to calculate the MST response—a quantitative parameter for the further analysis (p*K*_a_ determination).

Figure 2 shows the recorded fluorescence signals for FITC with the MST instrument in response to the change in pH value: before turning on the IR laser (top), and at three different MST powers corresponding to different temperature gradients formed as a function of time (bottom). The change of the initial fluorescence with increasing pH confirms the gradual change of the non-ionized form into the ionized form, already described in the literature [10]. In the case of changes in fluorescence after generation of a temperature gradient, a strong pH-dependent effect is also observed. The difference, however, is that the continuous change is recorded for buffers from 2 to 6, while buffer 1 breaks out of the trend and gives a stronger response than buffer 3, but weaker than 4. This situation is observed at both low, medium and high MST power. The magnitude of the change in fluorescence with time is correlated with the MST power. This dependence results directly from the thermal gradient, which the larger, the stronger the effect of thermophoresis and accompanying TRIC.

The clear trend recorded for buffers 2–6 indicates a likely relationship between the phenomenon of thermophoresis/TRIC and progressive deprotonation of FITC. It can also be assumed that the different response of buffer 1 (pH 5.38) results from the partial overlapping of two acid-base equilibria, the tested phenol (pH 6.62) and carboxyl (pH = 4.36) groups. In this scenario, the deprotonation of a carboxyl group may cause an opposite impact on MST response to that of the phenolic group. Accordingly, at lower pH values the increasing ionization of -COOH entails a decrease of MST response, and therefore, the thermophoresis/TRIC can seem to weaken with pH. On the other hand, when the -OH group starts to deprotonate, MST response grows and the thermophoresis/TRIC appears to escalate. Probably due to the fact that p*K*_a_ values of both groups are quite close to each other, at pH 5.38 the MST response could be concomitantly influenced by both effects, mainly by the incomplete ionization of -COOH. On that account, the data obtained at this pH deviates from the trend observed for the remaining buffers. In other words, the analytically-undesirable interference of two opposite effects was probably observed in the case of buffer 1. Based on the above assumption, the results obtained for buffer 1 were rejected, while the model allowing for the determination the p*K*_a_ value was fitted to the results obtained for buffers 2–6 (the hypothesis presented here is confirmed by the latest data collected in our laboratory for FITC in a wider pH range, which will be the subject of our next contribution aimed at the further methodology development).

### 2.2. Determining pK_a_ Values

As the MST response one should assume the value of fluorescence measured after heating the sample for 25 s (red band in Figure 2) divided by the fluorescence measured before heating (blue bar in Figure 2), expressed in per miles (‰). As suggested by the manufacturer of the MST instruments (NanoTemper Technologies, Munich, Germany), after such a long heating time, the thermophoretic effect is particularly strong, and it dominates the accompanying TRIC effect, which may indicate a direct relationship between the thermophoresis and the deprotonation process.

To fit the MST data in order to determine p*K*_a_ a sigmoidal Boltzmann curve was used [11] to describe the change in ionization degree of the molecule in aqueous solutions as a function of pH:

(1) $$y=\frac{A-B}{1+e^{\left(x-x_{0}\right)/dx}}+B$$

where *A* is the MST response (‰) standing for the non-ionized state asymptote, *B* is the MST response (‰) standing for the totally ionized state asymptote, *x*_0_ is the inflection point standing for the p*K*_a_ (50% ionization), and *dx* is a constant of 0.434 determining the pH range in which the ionization degree increases. This model worked well at finding p*K*_a_ values based on the electrophoretic data obtained by us in the past [12,13].

**Figure 3:**
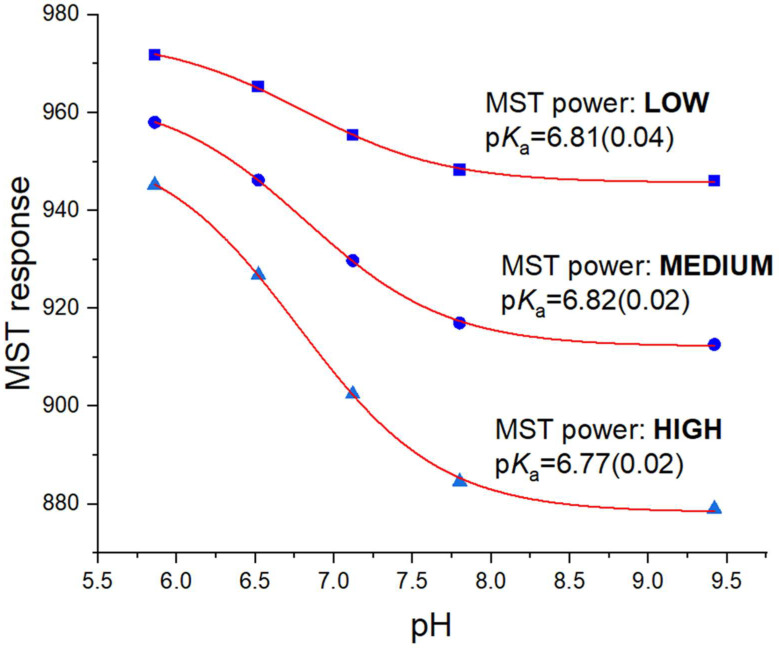
MST response as a function of pH at three IR laser powers, with non-linear fit and determined p*K*_a_ values. The error of p*K*_a_ determination is given in parentheses as the model fit error predicted by the Origin Pro 2020 software.

The obtained fits, together with the p*K*_a_ values are shown in Figure 3. As can be seen, the p*K*_a_ values for the low and medium MST powers are practically the same (the difference is within the fitting error), while the value for high power is slightly lower. However, one should take into account that with increasing IR laser power comes an increase in the temperature of the sample, whereas p*K*_a_ values depend on it. Thus, considering this uncertainty, it can be assumed that the p*K*_a_ values obtained with different instrumental setups are consistent. Therefore, as the manufacturer of the MST instrument advises, if it is possible to observe a given effect at different laser powers, it is better to choose the lowest possible one to avoid potential adverse effects related to excessive heating of the system.

**Figure 4:**
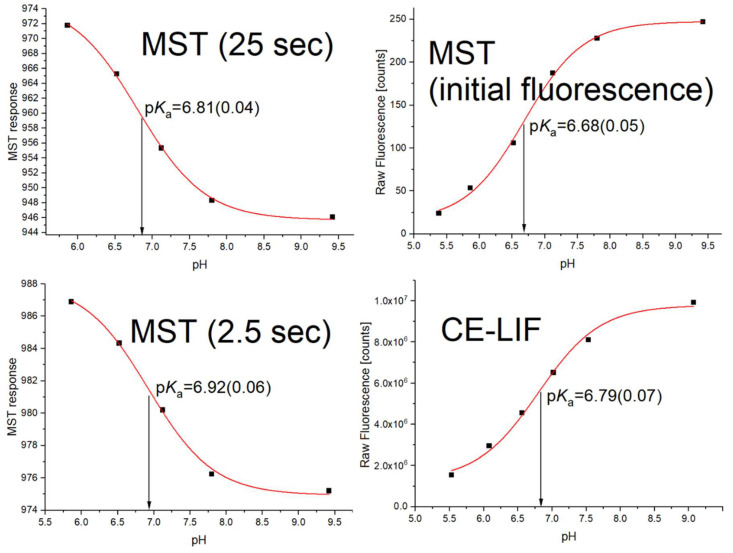
Comparison of the p*K*_a_ values determined on the basis of the thermophoresis/TRIC at different heating times (25 s and 2.5 s), and the reference values obtained by methods using fluorescence but independent of the thermophoresis/TRIC (MST and CE-LIF). The error of p*K*_a_ determination is given in parentheses. The same temperature (22 °C) was set up in the MST and CE-LIF instruments.

The p*K*_a_ value for FITC of about 6.8 is slightly higher than that reported in the literature for this compound in a similar ionic strength [10]. However, it should be taken into account that the acid-base equilibrium may change in the case of fluorophores as a result of their transition from the ground state to the excited state. This photoacidity effect is reflected in the observed change in p*K*_a_ upon excitation [14]. It depends on the mutual relationship of the deprotonation and fluorescence kinetics, as well as on the type of fluorophore and the environment, in particular on the ionic composition of the buffer. Considering the slight increase in p*K*_a_ for fluorescein upon excitation described already in the literature [15], this effect could also be observed for structurally related FITC. Therefore, to verify the accuracy of the determined p*K*_a_ value by means of MST, methods based on the change of fluorescence as a function of pH should be used when possible, obviously without creating a thermal gradient. For this purpose, the initial fluorescence values recorded by the MST instrument before switching on the IR laser were used (Figure 2 top), as well as the fluorescence values measured with an independent reference technique—the capillary electrophoresis using the laser-induced fluorescence detection (CE-LIF), characterized by high sensitivity and automatic injection of samples into the capillary that significantly reduces the potential error of fluorophore concentration variation (possible in MST due to manual pipetting of fluorophore to PCR tubes containing pH buffers). In addition, the aim was to check whether the heating time starting from switching on the IR laser may influence the p*K*_a_ value determined based on the phenomenon of thermophoresis/TRIC. The obtained data, together with the fitting of analogous models and the obtained p*K*_a_ values, are shown in Figure 4.

The p*K*_a_ values obtained by different methods are very similar, their span is about 0.2 pH unit. It is relatively small, especially considering the uncertainty of pH value measured with the pH-meter, which was not taken into account when estimating the error, which, as we suspect, is usually around 0.05–0.10 pH unit. Moreover, each approach was characterized by slightly different thermal conditions; the IR radiation and the application of high voltage in the case of CE-LIF are accompanied by different increases in sample temperature. In addition, thermostatic systems operate with different efficiency in the MST and CE instruments. In conclusion, taking into account that this is the very first attempt to estimate p*K*_a_ using thermophoretic data, the obtained agreement suggests a real possibility of using the MST technique for acidity analysis. However, this does not change the fact that although the obtained results provide the-proof-of-concept for this idea, further research is necessary to identify potential effects responsible for the accuracy of the method, explain unequivocally the deviations observed for buffer 1, develop a missing theoretical basis of the method (an equation linking MST response, pH and p*K*_a_), as well as examining other analytes and experimental conditions.

### 2.3. Thermodynamic Study

The last goal of this research was to find out whether the p*K*_a_ values obtained by the thermophoretic method at different temperatures will allow for the determination of the Van’t Hoff dependency and to estimate the standard enthalpy and entropy of the deprotonation process. As it is known [9], the Soret coefficient responsible for the observed MST response depends on the temperature not only due to the possible change in charge (if p*K*_a_ changes with temperature), but also other effects, for example hydration entropy. Due to the current lack of a proper theoretical model and detailed knowledge on the role of these effects in p*K*_a_ determination, it was assumed that any additional effects are similar in the entire working pH range, and thus they are compensated and do not significantly affect the position of the inflection point of the measured MST response versus pH. The methodology of p*K*_a_ determination based on electrophoretic mobility is based on a similar assumption [13] (the temperature dependence of electrophoretic mobility does not affect the obtained p*K*_a_ values obtained at different temperatures, so they are used to thermodynamic analysis).

The MST instrument was re-used, making additional measurements at 27, 32 and 37 °C. Fluorescence after switching on the IR laser was measured as before at 25th s. The obtained p*K*_a_ values, including the value obtained previously for the temperature of 22 °C, were converted into thermodynamic values ($pK_{a}^{T}$) using the Debye-Hückel model [16]:

(2) $$pK_{a}^{T}=pK_{a}-\log\gamma_{A^{z-1}}+\log\gamma_{HA^{z}}$$

where *γ* denotes activity coefficient of the particular species: protonated-*HA*, and deprotonated-*A*, with a given charge (*z* = −1 for *HA* and −2 for *A*). Activity coefficients were calculated as follows:

(3) $$-\log\gamma =\frac{Bz^{2}\sqrt{I}}{1+Ca\sqrt{I}}$$

where *B* and *C* are parameters dependent on dielectric constant and temperature, in water at 25 °C they are 0.509 and 0.33, respectively, *I* is the ionic strength (50 mM), while *a* denotes the radius of hydrated ion. The approximate value of *a* = 5 Å was used for calculations [16].

This was done to correct the p*K*_a_ value due to the fact that the measurements were carried out in 50 mM ionic strength buffers and not in a pure aqueous solution. The obtained thermodynamic acid dissociation constant values are shown in Table 1.

**Figure 5:**
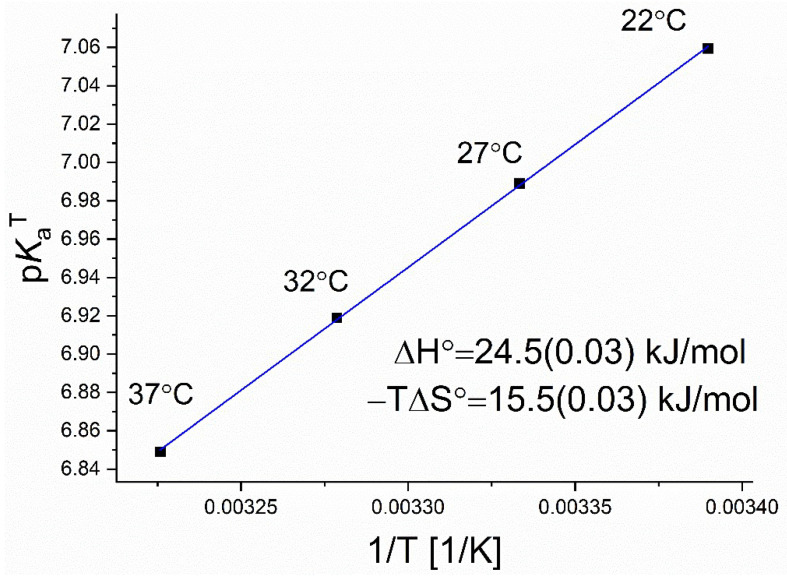
The dependence of the $pK_{a}^{T}$ values obtained using the MST method on the inverse absolute temperature, together with the values of thermodynamic functions obtained from the Van’t Hoff model. The temperature of 25 °C (298 K) was used to calculate the *T*Δ*S*° term.

Next, the $pK_{a}^{T}$ values were presented against the inverse absolute temperature to determine the thermodynamic functions based on the Van’t Hoff model (see Figure 5).

The Van’t Hoff model allows for the determination of the enthalpy and entropy contributions accounting for the acid-base dissociation process based on the equation:

(4) $$pK_{a}=\frac{\Delta H{}^{\circ}}{2.303RT}-\frac{\Delta S{}^{\circ}}{2.303R}$$

where *R* is the gas constant (8.3145 J·mol^−1^·K^−1^). The enthalpic (Δ*H*°) and entropic (−*T*Δ*S*°) terms were calculated from the slope and intercept, respectively. The temperature of 25 °C (298 K) was used to calculate the *T*Δ*S*° term [17].

The obtained values appear clearly on a straight line, which may confirm the accuracy of the method. A positive value of the deprotonation enthalpy was obtained which indicates the endothermic nature of the deprotonation/dissociation process. The entropic factors are also of great importance, constituting about 60% of the enthalpy value. Further thermodynamic analysis focused on structural effects responsible for the observed thermodynamic functions was not the aim of this study. However, the usefulness of the MST method for studying the thermodynamics of the acid dissociation process has been confirmed.

Nevertheless, as mentioned before, other effects related to temperature influencing the Soret coefficient [9] should be taken into account, and in the future, it is worth trying to develop a model that allows for the prediction of the thermophoretic properties of molecules on the basis of enthalpy and entropy factors determining the degree of ionization and, vice versa, to study their thermodynamics of deprotonation using the MST technique.

## 3. Discussion

The results we obtained clearly indicate that the acid-base equilibrium can be studied by the MST technique, due to the fact that deprotonation causes a strong thermophoretic response which is sufficient to determine the p*K*_a_ value. This method has a unique physicochemical basis and can be a perfect complement to other known technologies. Its advantages include ease of use, fast analysis time and the ability to carry out measurements in various media, including physiological ones. It is also suitable for thermodynamic analyzes that require the use of different temperatures. The necessity to rely on fluorescence cannot be eliminated at the moment, therefore the application of the presented methodology to determining p*K*_a_ values is restricted to fluorophores. Nevertheless, it can still serve as a good tool for performing investigations in several additional fields, including the following:

(a) supramolecular tuning of acid-base properties, i.e., determining changes in the p*K*_a_ value in systems enriched with the modifier (these may include macrocyclic compounds such as cyclodextrins, cucurbiturils, calixarenes, as well as various surfactants forming the micellar phase, macromolecules, e.g., proteins, and organic solvents) and research on the mechanism and thermodynamics of these changes. Investigation of this type is important, for instance for the development of strategies to increase the bioavailability of drugs [18]. The advantages of the MST technique (sensitivity, miniaturization, physiological conditions) may speak for its choice. Research can be carried out with the use of selected fluorophores, well described in terms of the structure-activity relationship, meeting the criteria of model compounds;
(b) basic research on the phenomenon of thermophoresis, its relationship with the acid-base equilibrium, aimed at developing a model that is missing for the moment [19,20], allowing for the prediction of the direction of thermophoresis based on the properties of the analyte (the mechanism responsible for the thermophoretic migration of ionized compounds remains unclear, moreover, the role of thermophoresis is discussed in the literature in creating a pH gradient [21,22], which was to play an important role in biogenesis as the initiation of biochemical reactions on the border of hot volcanic rock and cold water in the ancient ocean);
(c) as a very convenient model system for the development of new instrumental and methodological solutions using the phenomenon of thermophoresis. The new system demonstrated in this paper, the pH-dependent deprotonation of an analyte, gives a strong thermophoretic response, is simple in use and is inexpensive (the ligand used in affinity studies is replaced with an ordinary buffer). It can be used to develop on-line connections of MST with other techniques, e.g., developing the known CE-MST system [23], and alternative systems replacing fluorescence detection by another method, e.g., spectrophotometric.

## 4. Materials and Methods

The MST Monolith™ NT.115 instrument (NanoTemper Technologies GmbH, Munich, Germany) was used with blue range fluorescence excitation (465–490 nm) and 500–550 nm emission band pass filter. The standard capillaries dedicated to Monolith NT.115 were used (NanoTemper Technologies). The excitation source (LED) power was 2%, the IR laser power was set to low, medium or high, as described later in the text. The system was thermostated at 22 °C and, in the case of thermodynamic analysis, also at 27, 32 and 37 °C. The FITC supplied by Sigma-Aldrich (St. Louis, MO, USA) was used as the analyte. FITC was dissolved in an aqueous buffer of a given pH at a final concentration of 25 ng/mL, and then manually introduced into the MST capillaries by immersing them in the solution. For comparing the p*K*a values obtained with the MST, the PA 800 plus Capillary Electrophoresis instrument was used (Beckman-Coulter, Brea, CA, USA), equipped with the laser-induced fluorescence detector (CE-LIF). The analysis was carried out at the wavelengths of 488 nm-excitation, and 520 nm-detection. The analytical signal was the peak height at the maximum of electrophoretic peak of FITC measured in the electropherogram. The temperature and separation voltage were set up at 22 °C and 20 kV, respectively. The unmodified bare fused-silica capillary was used (Beckman-Coulter). It was of 60.0 cm total length, 50.0 cm effective length, and of 50 µm internal diameter. Between runs the capillary was rinsed with 0.1 M NaOH for 1 min, and running buffer for 2 min. Before the first use of the capillary at a working day: methanol for 10 min, 0.1 M HCl for 3 min, deionized water for 5 min, 0.1 M NaOH for 10 min, and running buffer for 10 min were applied. For the fresh capillary conditioning, the latter sequence was used but the duration of each individual step was doubled. The pressure applied equaled to 137.9 kPa (20 psi). Sample injection was conducted using the forward pressure of 3.45 kPa (0.5 psi) for 5 s. An acetate buffer (CH_3_COOH/CH_3_COONa) was used in the range of pH up to 6, phosphate buffer (NaH_2_PO_4_/Na_2_HPO_4_) in pH range up to 9 and borate (Na_2_B_4_O7·10H_2_O) at pH above 9. All buffers had the same ionic strength of 50 mM. All measurements with the use of MST and CE-LIF instruments were done in triplicate, all parameter values were subsequently averaged for the further analysis. All reagents except FITC were supplied by Avantor Performance Materials Poland. SA (Gliwice, Poland).

## 5. Conclusions

The presented results show for the first time that a relationship between the change in the thermophoresis/TRIC and deprotonation can be used to accurately estimate the acidity constant. The obtained p*K*_a_ values are consistent with the reference methods, which proves that this may become a new application of the MST technique used so far for supramolecular target-ligand affinity analysis. In such a case, in analogy, the deprotonated analyte plays the role of the free target, hydrogen ions play the role of a ligand with a concentration determined by the pH value, and the protonated (undissociated) analyte plays the role of the target-ligand complex. This work is only the-proof-of-concept of the new idea, and further research is required to determine the actual analytical performance and usefulness of the method more reliably. The biggest limitation is reliance on the phenomenon of fluorescence. Nevertheless, in the future, it is likely that new technologies will be developed based on the phenomenon of thermophoresis, but using a more universal method of detecting changes in analyte concentration under the influence of temperature. At present, this method can be used to study the acid-base dissociation of selected groups of molecules and its thermodynamics; investigate changes of acidity caused by addition of various modifiers, e.g., macrocyclic compounds, surfactants, macromolecules and nonaqueous solvents; discover yet unknown foundations of the thermophoresis process and its relationship with ionization; and serve as an easy to use model system to develop MST technology and couple it with other techniques. Therefore, this work may open up completely new research opportunities.

## Figures and Tables

**Table 1:** The p*K*_a_ values of FITC determined at various temperatures using the MST technique, before and after the correction of the ionic strength effect.

Temp. [°C]	p*K*_a_ (IS = 50 mM)	$pK_{a}^{T}\;(\mathrm{IS}=0\;\mathrm{mM})$
22	6.81 (0.04)	7.06
27	6.74 (0.01)	6.99
32	6.67 (0.06)	6.92
37	6.60 (0.05)	6.85

## Data Availability

Raw data supporting the manuscript content are publicly available using the link: (https://ruj.uj.edu.pl/xmlui/handle/item/286417; DOI:10.26106/p7x8-y113) (accessed on 15 December 2021).
